# T65. EVALUATING PATTERNS OF SEMANTIC AND EXECUTIVE DYSFUNCTION IN SCHIZOPHRENIA: A CLUSTER ANALYSIS APPROACH

**DOI:** 10.1093/schbul/sby016.341

**Published:** 2018-04-01

**Authors:** Eric Tan, Denny Meyer, Erica Neill, Caroline Gurvich, Susan Rossell

**Affiliations:** 1 Swinburne University; 2 University of Melbourne; 3 The Alfred Hospital and Monash University, Swinburne University

## Abstract

**Background:**

Semantic and executive dysfunction are among the most prominent of the cognitive impairments in schizophrenia. Using a cluster analysis (CA) approach, the primacy of semantic and executive dysfunction and their relationship to psychopathology was examined in a two-step investigation.

**Methods:**

In Study One, 76 schizophrenia/schizoaffective disorder (SZ) patients completed three semantic (category fluency productivity, category errors, Hopkins Verbal Learning Test) and three executive function (inhibition, switching, verbal fluency) measures. Three groups were predicted: semantic-dominant (SD), executive-dominant (ED) and mixed. In Study Two, 52 SZ patients and 48 healthy controls completed the MATRICS Consensus Cognitive Battery (MCCB) alongside the previous semantic/executive battery.

**Results:**

For Study 1, the CA results confirmed the first two specific groups but revealed a third group unimpaired in both domains (UN). Positive and negative symptoms did not differ between all groups. For Study 2, the CA results confirmed the presence of the same three groups: SD, ED and UN. One-way ANOVAs confirmed that MCCB overall cognitive scores for UN group were significantly higher compared to the SD and ED groups, which did not differ from each other; however, all three clinical groups still performed significantly worse than healthy controls. Psychopathology again did not differ between the three clinical groups.

**Discussion:**

The findings confirm semantic and executive dysfunction as two main areas of cognitive impairment in SZ while also affirming the presence of cognitively impaired patients without these two primary deficits. Symptomatology patterns do not appear to differ between cognitive impairment profiles, highlighting the complexity of symptomatology mechanisms and cognitive deficits being a discrete entity within the illness. These conclusions have implications for the nosology of schizophrenia and the delivery of cognition-based therapies.

